# Unsupervised invariance learning of transformation sequences in a model of object recognition yields selectivity for non-accidental properties

**DOI:** 10.3389/fncom.2015.00115

**Published:** 2015-10-07

**Authors:** Sarah M. Parker, Thomas Serre

**Affiliations:** ^1^Department of Cognitive, Linguistic, and Psychological Sciences, Brown UniversityProvidence, RI, USA; ^2^Brown Institute for Brain SciencesProvidence, RI, USA

**Keywords:** inferotemporal cortex, ventral stream, Hmax, invariance, object constancy, object recognition, learning

## Abstract

Non-accidental properties (NAPs) correspond to image properties that are invariant to changes in viewpoint (e.g., straight vs. curved contours) and are distinguished from metric properties (MPs) that can change continuously with in-depth object rotation (e.g., aspect ratio, degree of curvature, etc.). Behavioral and electrophysiological studies of shape processing have demonstrated greater sensitivity to differences in NAPs than in MPs. However, previous work has shown that such sensitivity is lacking in multiple-views models of object recognition such as Hmax. These models typically assume that object processing is based on populations of view-tuned neurons with distributed symmetrical bell-shaped tuning that are modulated at least as much by differences in MPs as in NAPs. Here, we test the hypothesis that unsupervised learning of invariances to object transformations may increase the sensitivity to differences in NAPs vs. MPs in Hmax. We collected a database of video sequences with objects slowly rotating in-depth in an attempt to mimic sequences viewed during object manipulation by young children during early developmental stages. We show that unsupervised learning yields shape-tuning in higher stages with greater sensitivity to differences in NAPs vs. MPs in agreement with monkey IT data. Together, these results suggest that greater NAP sensitivity may arise from experiencing different in-depth rotations of objects.

## 1. Introduction

Invariant object recognition is a notoriously challenging computational problem (Marr, 1982). Our visual system has to deal with large intra-class variations owing to the effect of 2D and 3D transformations (including translation, scaling and rotation) because small changes in an object's 3D view may yield large changes on its 2D projection on our retinas. Yet, despite these large intra-class variations, primates are capable of robustly and effortlessly recognizing objects (Thorpe et al., 1996), vastly outperforming the best existing computer vision systems.

**Figure 1:**
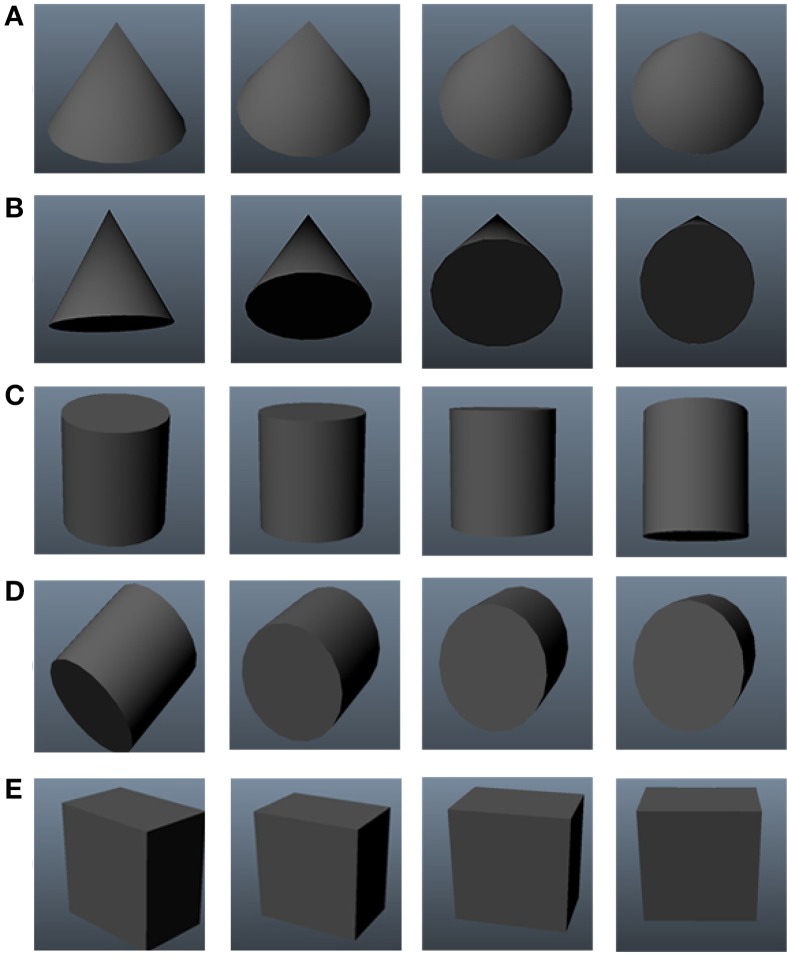
**Representative appearance changes undergone by objects during out-of-plane rotations**. Variations of metric properties here include: **(A)** increasing angle at a point and **(B)** increasing size, shape and curvature of cross section of a cone **(C)** increasing size, shape and curvature of cross section of a cylinder **(D)** decreasing length of a cylinder and **(E)** decreasing area of cross-section and increasingly skewness of the edges of a cube.

Object constancy requires the development of visual representations that remain stable across object transformations (Földiák, 1998). In particular, one may distinguish between those object properties that will remain stable across changes in viewpoint and those that will not (see Figure 1, for an illustration). Properties such as the degree of curvature of an object's contours, its length, or the amount of expansion of a cross section are examples of properties that will be affected by changes in viewpoint. Conversely, there also exist qualitative shape properties that remain stable across changes in viewpoint, e.g., whether an edge is straight or curved, whether a surface is convex or concave, or whether a cross section ends at a point vs. a side. These qualitative properties are known as non-accidental properties (NAPs) and need to be contrasted with their quantitative counterparts known as metric properties (MPs).

There is a long history of studies related to NAPs in computational vision (see Lowe, 1984, for review): From a theoretical point of view, a visual system needs to focus on the detection of image structures that are unlikely to have arisen by accident. For instance, the probability of a curved edge to appear straight because of projection is extremely small and would happen as an “accident” of viewpoint (Richards et al., 1996). The stability of NAPs over viewpoints makes them useful for achieving object constancy. Indeed, NAPs have been the focus of a prominent psychological theory of object recognition called the Recognition-by-Components (RBC) theory (Biederman, 1987). Briefly, this *structural-description* theory states that the visual system may encode a finite visual vocabulary of basic 3D shapes called geons. These geons can be differentiated on the basis of differences in NAPs, and generic object categories can be represented as compositions of geons. This theory has motivated the design of a number of experimental studies and it is now relatively well established that our visual system exhibit greater sensitivity to differences in NAPs compared to MPs (see Biederman, 2007, for review).

Behaviorally, it has been shown that participants can more accurately distinguish between two objects that differ along an NAP vs. an MP (Biederman and Bar, 1999). Furthermore, when trained to recognize novel object categories where two NAPs (the degree of curvature and the degree of parallelism) are systematically varied, adult participants are more likely to treat a change in NAP as categorical (as opposed to within-category variation) compared to a similar change in MP (Abecassis et al., 2001). When a more sensitive paradigm is employed, preschool children, like adults, find it easier to discriminate NAPs vs. MPs (Amir et al., 2014). In addition, both adults and 4 month olds exhibit a saccadic preference for NAPs vs. MPs (Amir et al., 2011).

The neural basis of NAP selectivity was more directly studied by Kayaert et al. (2003) who recorded neuronal responses in the inferior temporal cortex (ITC) of the macaque. It was shown that neural responses are more strongly modulated by changes in NAPs than by equally large pixel-wise changes in MPs (Kayaert et al., 2003).

Further work later showed that such increased NAP sensitivity is incompatible with *multiple-views* models of object recognition such as the Hmax (see Riesenhuber and Poggio, 1999; Serre, 2014, for reviews), which assume that shape processing is based on broadly-tuned neuronal populations with distributed symmetric bell-shaped tuning: Shape-tuned units in these models are modulated at least as much by differences in MPs as in NAPs (Amir et al., 2012). It remains an open question—if and how—Hmax can be modified to account for the increased NAP sensitivity found both behaviorally and electrophysiologically.

Here, we test the hypothesis that mechanisms for learning transformation sequences may increase the model sensitivity to differences in NAPs vs. MPs. Given that MP changes result in part from generic object transformations (3D rotation), and given the focus of the original model on 2D transformations, we reasoned that learning invariances to natural object transformations should yield a decrease in the sensitivity of model units to MPs compared to NAPs (see Tarr and Kriegman, 2001, for a similar argument). To test our hypothesis, we created a database of video sequences with objects slowly rotating in depth in an attempt to mimic sequences viewed during object manipulation by young children during early developmental stages (**Figure 3**).

Several algorithms have been proposed for learning transformation sequences (e.g., Perrett et al., 1984; Foldiak, 1991; Hietanen et al., 1992; Wallis et al., 1993; Einhäuser et al., 2002; Wiskott and Sejnowski, 2002; Spratling, 2005; Stringer et al., 2006; Masquelier et al., 2007). Here, we consider a simple form of sequence learning via a “temporal pooling” mechanism similar to that used in the Hierarchical Temporal Memory algorithm (Hawkins and Blakeslee, 2004). The basic idea is to incorporate invariance pooling mechanisms in intermediate stages of the Hmax to include more generic object transformations (such as 3D rotation).

**Figure 2:**
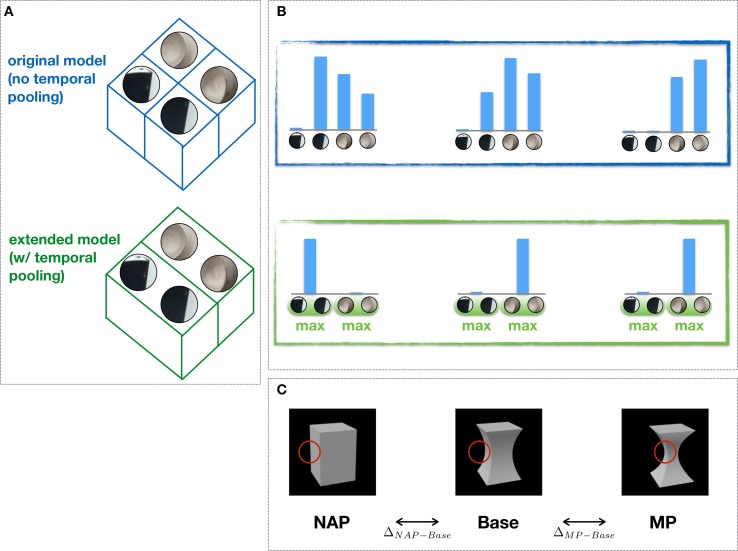
**Feature columns, invariance to object transformations and NAP sensitivity. (A)** Feature columns in the extended (bottom) vs. the original (top) model (i.e., w/ and w/o temporal pooling). One of the key computational mechanisms in the Hmax builds on the proposal by Hubel and Wiesel (1962) to achieve tolerance of 2D transformations via a selective pooling mechanism (at the level of complex cells) over afferent units with the same preferred selectivity (feature) but slightly different positions and scales (not shown). Here, we propose a simple extension of this idea to include a more general form of pooling, i.e., over a transformation sequence of the preferred stimulus learned through visual experience. This pooling is done within feature columns which include different views of the same feature learned from object transformation sequences. **(B)** Shown are the corresponding patterns of (column) activity for the original and the extended model. **(C)** Sample stimuli used to probe the selectivity for MP (Δ_*MP*−*Base*_) vs. NAP (Δ_*NAP*−*Base*_) changes from a Base stimulus as done in Kayaert et al. (2003). Whereas the original model fails to exhibit any sensitivity to NAP vs. MP changes (Δ_*NAP*−*Base*_ ≈ Δ_*MP*−*Base*_), the extended model exhibits greater tolerance to object transformation through the “temporal pooling” mechanism and, as a result, greater sensitivity to NAP vs. MP changes (Δ_*NAP*−*Base*_ > Δ_*MP*−*Base*_). Shown in red is the hypothetical stimulus location driving the unit response.

In the original model, IT-like units in the the last stage are organized in feature columns (Figure 2A) modeled after those found in cortex (Tanaka, 2003): Each feature column is characterized by its tuning for a distinct visual feature over a range of positions and scales. Feature selectivity is learned from individual object views (Serre et al., 2007b) and each column activity reflects the degree of similarity between an input stimulus and the corresponding preferred feature. Assuming *N* feature columns, the resulting population activity encodes an input stimulus as an *N*-dimensional pattern of activity (Figure 2B). The difference in the pattern of activity associated with two distinct input stimuli reflects the visual dissimilarity between the two stimuli and does not distinguish between an MP vs. NAP change (Δ_*MP*−*Base*_ ≈ Δ_*NAP*−*Base*_; Figure 2C).

In the extended model, feature columns include multiple views of the same feature sampled from short object transformation sequences (~300 ms). The responses of features within a column are then combined via a max operation (as done in the original model for invariance to position and scale; Figures 2A,B). Such unsupervised learning mechanism is consistent with both human behavioral (Wallis and Bülthoff, 2001; Cox et al., 2005) and nonhuman primate (Li et al., 2008, 2010) studies which suggest that tolerance to object transformations is at least partly supported by the natural temporal contiguity of visual experience. As we will show, the proposed pooling mechanism yields a visual representation which exhibits greater tolerance to object transformations and, as a result, a greater sensitivity for NAP compared to MP changes (Δ_*MP*−*Base*_ < Δ_*NAP*−*Base*_) in agreement with neurophysiological data.

## 2. Materials and methods

### 2.1. Video database

**Figure 3:**
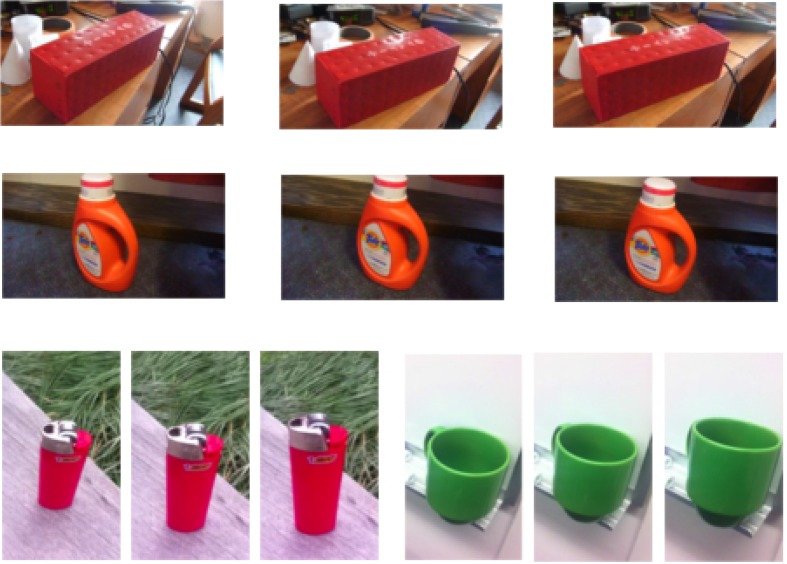
**Representative frames sampled from a collected video database of everyday objects undergoing 3D transformations (i.e., combination of translation, scaling, and in-depth rotation)**.

We used a consumer-grade camera to collect short video sequences (30 Hz) with the aim to mimic object manipulations (Figure 3). Everyday objects were placed in diverse environments and the camera was moved slowly around the object to create 3–5 s long videos of the object undergoing a transformation (combination of small translation, scaling, and in-depth rotation). The video database included 12 common objects routinely found in a dorm room with at least 20 video sequences per category for a total of about 240 video sequences. For each category, the object background, initial viewpoint, and magnitude of the rotation was varied as much as possible.

### 2.2. The Hmax model

Here, for convenience, we used a somewhat simplified implementation of the Hmax, which includes only four processing stages (Serre et al., 2007b). We only very briefly review the model architecture as details of the implementation have been described elsewhere (see Serre et al., 2007b; Serre, 2014, for details) and source code for the model is publicly available at: http://serre-lab.clps.brown.edu/resources.

The Hmax model of object recognition combines a hierarchical build-up of invariance and selectivity (inspired by Fukushima, 1980) with the idea of multiple-views (view-based) recognition of 3D objects (Riesenhuber and Poggio, 1999, 2000). Over the years, several related hierarchical models have been developed (Mel, 1997; Wallis and Rolls, 1997; LeCun et al., 1998; Riesenhuber and Poggio, 1999; Ullman et al., 2002; Amit and Mascaro, 2003; Wersing and Köerner, 2003; Masquelier and Thorpe, 2007; Mutch and Lowe, 2008; Jarrett et al., 2009; Pinto et al., 2011; Saxe et al., 2011). We focus here on the Hmax because the underlying parameters of the architecture were explicitly derived from available neuroscience data and because this was the model originally tested for NAP modulation and compared against IT data by Amir et al. (2012). Without loss of generality, we expect related models to exhibit similar trends.

Each processing stage in the Hmax model is organized in columns. Each column contains a complete dictionary of *S* unit selectivities for that particular layer. For instance, a column in the first *S*_1_ stage (modeled after simple cells in striate cortex; see Lades et al., 1993, for an early system using Gabor filters for face recognition) contains a complete range of orientation and spatial frequency tuning and a column of *S*2 units (corresponding to units in intermediate areas of the ventral stream of the visual cortex) to a complete dictionary of shape-tuned units (see later). Simple units pool over afferent units using a Gaussian-like tuning operation. That is, the response *y* of a simple unit, receiving the pattern of inputs **x** from the previous layer is given by *y* = exp − γ||**w** − **x**||^2^, where γ defines the sharpness of the tuning around the preferred stimulus of the unit corresponding to the weight vector **w**. These columns are then replicated at different positions and scales, which is the key mechanism by which the model gains its tolerance to 2D transformations (position and scale) at the level of *C* units. The pooling operation at the level of complex units is a max operation over afferent units. That is, the response *y* of a complex unit from the previous layer is given by *y* = max_j∈pool_*x_j_*. The parameters governing the invariance properties of the *C* units (i.e., the size of the pooling range over position and scale) is constrained by available physiology data (Serre et al., 2007a).

In the original model, the only learning that takes place is at the level of the dictionary of *S*_2_ units. This is done via an *imprinting* learning rule whereby during the training procedure, units store patterns of neural activity associated with the presentation of patches of natural scenes that are presented in their receptive field (see Serre et al., 2007b, for details). More sophisticated algorithms have been proposed for learning intermediate visual features (e.g., Shams and von der Malsburg, 2002; Ullman et al., 2002; Masquelier and Thorpe, 2007; Hu et al., 2014). Here, without loss of generality, we used the simple imprinting learning rule to stay as faithful as possible to the original model but it is expected that other algorithms would yield qualitatively similar results.

### 2.3. Measuring NAP selectivity

**Figure 4:**
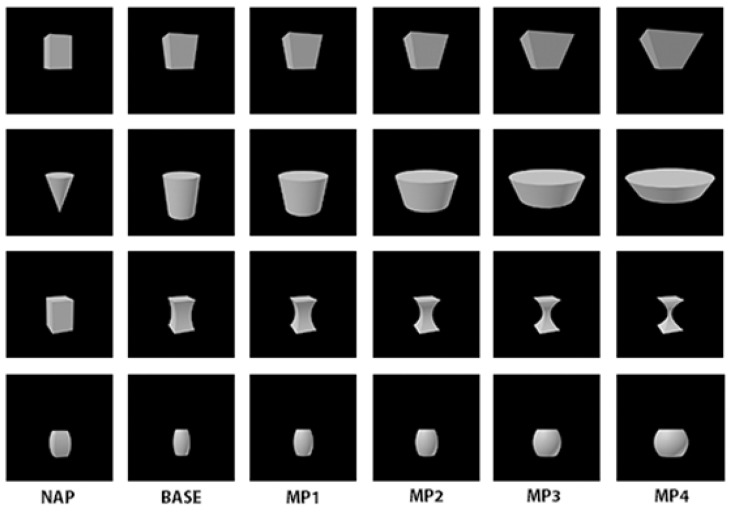
**Sample stimuli from the study by Kayaert et al. (2003)**. The column labeled BASE corresponds to a reference image. The column labeled NAP corresponds to a transformation of the base image where an NAP was changed. MP1, MP2, MP3, and MP4 correspond to a transformation of the base image with an MP of increasing magnitude.

Here we conducted *in silico* experiments on the Hmax model with the aim to mimic the experimental methods described in the original studies (Kayaert et al., 2003; Amir et al., 2012) as closely as possible. The stimulus set consisted in the 36 basic shapes used in Kayaert et al. (2003). Each of the 36 stimuli exhibited five level of variations along a single dimension: four metric variations of increasing amplitude (denoted MP1–MP4) and one non-accidental variation (denoted NAP). The NAP variation was calibrated so that the resulting change from the base shape (measured by the euclidean distance directly on pixel intensities) was equal or less than the change associated with MP2. Sample stimuli are shown on Figure 4. The NAP/MP percent modulation for model units was computed using the same formula as described in the original study by Kayaert et al. (2003): (response basic shape—response to object variation)/(response basic shape)^*^100.

## 3. Results

**Figure 5:**
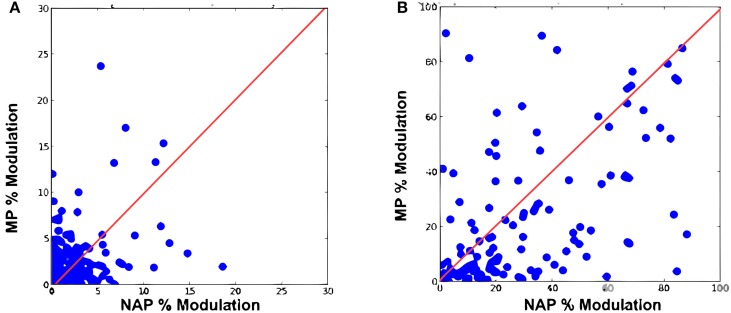
**Percent modulation of ***C***_2_ units from the base image to MP2 vs. percent modulation of ***C***_2_ units from the base image to NAP for (A) original Hmax (B) the extended model**.

We first reproduced the results by Amir et al. (2012) demonstrating that the original Hmax failed to exhibit a greater sensitivity for NAPs vs. MPs. We trained a baseline model with the object video dataset (Section 2; Figure 3). As in the original electrophysiology study, units were selected based on their visual responsiveness to the base images in the stimulus dataset used for electrophysiology (see Kayaert et al., 2003, for details) which yielded 243 NAP-MP comparisons. For each model unit, we computed the NAP and MP percent modulation for its preferred stimulus (Section 2). Figure 5A shows the MP percent modulation vs. NAP percent modulation for each unit in the original model. We found an average of 20% NAP modulation, compared to an average 22% MP modulation from the base object. A wilcoxon signed-rank test confirmed no significant NAP vs. MP modulation (*p* = 0.76). Overall, only 49% of the units had a greater NAP modulation, as compared to the 63% found in IT (Kayaert et al., 2003).

We then proceeded to extend the model to learning invariances from transformation sequences. In the original model, IT-like units are organized in feature columns whose selectivity is determined by a simple imprinting learning rule (Section 2). Each feature column (*C*_2_ unit) is hard-coded by considering afferent (*S*_2_) units tuned to the same preferred feature but with receptive fields at different locations (and scales), yielding a visual representation which is tolerant to 2D transformations. However, no mechanism for invariance to 3D transformations is present in the original model yielding a “salt-and-pepper” organization of feature columns for changes in viewpoint (Figure 2A).

Here, we extended the invariance pooling mechanism to also include different views of a feature undergoing a 3D transformation during a relatively small (~300 ms) time window. This was done by considering feature columns which include multiple units with a selectivity for different views of the same feature occurring in close temporal proximity.

Visual responsiveness for this new set of *C*_2_ model units was assessed as for the original model which yielded 159 NAP-MP comparisons. As shown on Figure 5B, this model extension yielded a dramatic increase in NAP vs. MP modulation with an average 35% NAP modulation vs. a 24% MP modulation. A Wilcoxon test showed a significant modulation for NAP vs. MP (*p* < 0.01). We further observed that 71% of the new model units were now more strongly modulated by a change in NAP vs. MP. As seen in Figure 5B, the majority of data points now fell below the diagonal, illustrating a greater sensitivity to NAP change. Table 1 summarizes these findings and provides a comparison to IT data reported in Kayaert et al. (2003).

**Table 1:** **Comparison between IT Data (Kayaert et al., 2003), the original as well as the extended Hmax**.

	**% NAP Modulation from base**	**% MP Modulation from base**	**Sample size**	**Wilcoxon *p*-value**	**% units NAP>MP Modulation**
IT Data	33	21–26	*n* = 243	*p* < 2e-06	63
Original Hmax	20	22	*n* = 243	*p* = 0.7645	49
Extended Hmax	35	24	*n* = 159	*p* = 1.2e-05	71

*Sample sizes correspond to the number of NAP-MP comparisons as done in the original study*.

Interestingly, we also found that learning transformation sequences yielded a significant improvement in object recognition classification accuracy over changes in viewpoint. We used the scikit-learn toolbox (Pedregosa et al., 2011) to train and test a multi-class linear SVM on the original and extended model outputs using a random split procedure of the video dataset (*n* = 15). The regularization parameter was optimized using a cross-validation procedure. We found an overall significantly higher accuracy for the extended model (95.2 ± 2.1%, chance level: 8.3%) vs. the original model (85.6 ± 1.8%, *p* < 0.01) suggesting that the proposed unsupervised invariance learning algorithm does indeed yield a model with greater generalization to changes in viewpoint.

## 4. Discussion

We have described a simple extension of a hierarchical model of object recognition (Hmax) which enables the network to learn transformation sequences. The original model includes mechanisms for building tolerance to 2D transformations (position and scale). We have shown that the proposed extension yields a model with better generalization capability for more complex transformation sequences which also include 3D rotations. Most importantly, we have shown that the resulting model exhibits greater sensitivity for NAPs vs. MPs in better agreement with IT data (Kayaert et al., 2003).

While our study has focused on the Hmax model, we expect our main results to apply broadly to the general class of feedforward hierarchical models (see Serre, 2014, for review). Despite differences in their specific wiring and detailed architecture, tolerance to object transformations in these models arise from Hubel-Wiesel types of pooling mechanisms and we thus expect our results to generalize to this broad class of models. Similarly, we also expect different learning rules to yield qualitatively similar results. While the present learning rule yielded NAP modulation in excellent agreement with IT data, it remains an open question whether other learning rules would provide similar or better fit to data.

Overall, our study suggests that the greater sensitivity for NAPs over MPs, as reported in several behavioral and electrophysiological studies (see Biederman, 2007, for review) may be driven by computational mechanisms for invariant object recognition.

## Author contributions

SP and TS conceived the research. SP performed the research. SP and TS wrote the manuscript and approved the final version for submission.

## Funding

This work was supported by DARPA young faculty award [grant number YFA N66001-14-1-4037] and NSF early career award [grant number IIS-1252951]. Additional support was provided by ONR [grant number N000141110743].

### Conflict of interest statement

The authors declare that the research was conducted in the absence of any commercial or financial relationships that could be construed as a potential conflict of interest.
